# Supplementary material for: Iterative improvement in the automatic modular design of robot swarms
Source: PeerJ Comput Sci. 2020 Dec 7;6:e322. doi: 10.7717/peerj-cs.322 (PMC7924708; doi:10.7717/peerj-cs.322)
Supplement: Supplemental Information 3 [file peerj-cs-06-322-s003.zip › argos3/doc/api/standalone/a00404_source.html]

ARGoS: core/utility/signal\_processing.cpp Source File


- Main Page
- Related Pages
- Namespaces
- Classes
- Files

- File List
- File Members

# core/utility/signal\_processing.cpp

Go to the documentation of this file.

```
00001 #include "signal_processing.h"
00002 
00003 namespace argos {
00004 
00005    /****************************************/
00006    /****************************************/
00007    
00008    CStats::CStats() :
00009       m_unCounter(0),
00010       m_fMean(0.0f),
00011       m_fSumOfSquareDiff(0.0f) {}
00012 
00013    /****************************************/
00014    /****************************************/
00015 
00016    void CStats::Append(Real f_value) {
00017       /*
00018        * Algorithm taken from:
00019        * http://en.wikipedia.org/wiki/Algorithms_for_calculating_variance#Online_algorithm
00020        */
00021       ++m_unCounter;
00022       Real fDelta = f_value - m_fMean;
00023       m_fMean += fDelta / static_cast<Real>(m_unCounter);
00024       m_fSumOfSquareDiff += fDelta * (f_value - m_fMean);
00025    }
00026 
00027    /****************************************/
00028    /****************************************/
00029    
00030    Real CStats::GetVariance() const {
00031       return m_unCounter > 1 ?
00032          (m_fSumOfSquareDiff / static_cast<Real>(m_unCounter - 1))
00033          :
00034          0.0f;
00035    }
00036 
00037    /****************************************/
00038    /****************************************/
00039 
00040    CRCLowPassFilter::CRCLowPassFilter(Real f_smoothing_factor) :
00041       m_fSmoothingFactor(f_smoothing_factor),
00042       m_bInitialized(false) {}
00043    
00044    /****************************************/
00045    /****************************************/
00046    
00047    Real CRCLowPassFilter::Filter(Real f_input) {
00048       if(m_bInitialized) {
00049          m_fPreviousOutput = f_input + m_fSmoothingFactor * (m_fPreviousOutput - f_input);              
00050       }
00051       else {
00052          m_fPreviousOutput = f_input;
00053          m_bInitialized = true;
00054       }
00055       return m_fPreviousOutput;
00056    }
00057 
00058    /****************************************/
00059    /****************************************/
00060    
00061    void CRCLowPassFilter::Reset() {
00062       m_bInitialized = false;
00063    }
00064 
00065    /****************************************/
00066    /****************************************/
00067    
00068 }
```

---

Generated on 10 Jul 2018 for ARGoS by 
 1.6.1
